# Comparative Performance Analysis of a Simplified Curzon-Ahlborn Engine

**DOI:** 10.3390/e20090637

**Published:** 2018-08-25

**Authors:** Ricardo T. Páez-Hernández, Juan Carlos Chimal-Eguía, Delfino Ladino-Luna, Juan Manuel Velázquez-Arcos

**Affiliations:** 1 Área de Física de Procesos Irreversibles, Departamento de Ciencias Básicas, Universidad Autónoma Metropolitana, U-Azcapotzalco. A. San Pablo 180, Col. Reynosa, Ciudad de México CP 02200, Mexico; 2 Laboratorio de Simulación y Modelado, Centro de Investigaciónen Computación, Instituto Politécnico Nacional, Av. Juan de Dios Batiz s/n UP Zacatenco, Ciudad de México CP 07738, Mexico

**Keywords:** Maximum Power Output (MP) regime, Maximum Power Density (MPD) regime, Maximum Efficient Power (MEP) regime, efficiency, Finite Time Thermodynamics (FTT)

## Abstract

This paper presents a finite-time thermodynamic optimization based on three different optimization criteria: Maximum Power Output (MP), Maximum Efficient Power (MEP), and Maximum Power Density (MPD), for a simplified Curzon-Ahlborn engine that was first proposed by Agrawal. The results obtained for the MP are compared with those obtained using MEP and MPD criteria. The results show that when a Newton heat transfer law is used, the efficiency values of the engine working in the MP regime are lower than the efficiency values (τ) obtained with the MEP and MPD regimes for all values of the parameter $\tau =T_{2}/T_{1}$, where $T_{1}$ and $T_{2}$ are the hot and cold temperatures of the engine reservoirs $\left(T_{2}<T_{1}\right)$, respectively. However, when a Dulong-Petit heat transfer law is used, the efficiency values of the engine working at MEP are larger than those obtained with the MP and the MPD regimes for all values of τ. Notably, when 0<τ<0.68, the efficiency values for the MP regime are larger than those obtained with the MPD regime. Also, when 0.68<τ<1, the efficiency values for the aforementioned regimes are similar. Importantly, the parameter τ plays a crucial role in the engine performance, providing guidance during the design of real power plants.

## 1. Introduction

The concept of Carnot’s efficiency is one of the cornerstone of thermodynamics. It serves as the upper bound for the heat engine efficiency between two heat reservoirs; however, when the engines are operating infinitely slower, this is obviously unrealistic. From the second half of the 20th century, research has focused on identifying performance limits of thermodynamic processes and optimizing thermodynamic cycles. Novikov [1], Chambdal [2], and Curzon-Ahlborn [3] were the first to extend the Carnot cycle, considering the irreversibilities of finite time, to show that a Carnot engine with heat resistance in its reservoirs has a maximal power production, and this maximum thermal efficiency can be described by $\eta_{CA}=1-\sqrt{T_{2}/T_{1}}=1-\tau$. From the pioneer work of Curzon-Ahlborn, a new branch of irreversible thermodynamics appeared called Finite Time Thermodynamics (FTT), which have inspired many articles that focused on power optimization or minimization of fixed costs for heat engines, endoreversibles, non-endoreversibles, and finite size constrains under various heat transfer laws, including linear and non-linear, among others [4,5,6,7,8,9,10]. Extensive details about the FTT background can be found in Bejan [11] and Cheng et al. [12]. However, the above mentioned works did not consider the effect of engine size related to investment cost. To incorporate the effects of size in performance analysis, Sahin et al. [13] introduced a new optimization criterion called the Maximum Power Density (MPD) analysis. Using this criterion, some authors investigated the optimal performance of heat engines. For instance, Sahin et al. [14] found the efficiency of a Joule-Brayton engine at maximum power density, Kodal et al. [15] analyzed the comparative performance of irreversible Carnot heat engines under maximum power density and maximum power conditions, and Chen et al. [16] analyzed the efficiency of an Atkinson engine at maximum power density. Later, Yilmaz et al. [17], in order to consider the effects on the design of heat engines, introduced the Maximum Efficient Power (MEP) criterion, as the multiplication of power by cycle efficiency. This criterion not only considers the power output but also the cycle efficiency, which was successfully applied to the Carnot, Brayton, and diesel engines, among other systems [18,19].

This paper presents a Maximum Power Output (MP), Maximum Efficient Power (MEP), and Maximum Power Density (MPD) performance analysis for a simplified version of the Curzon-Ahlborn engine proposed by Agrawal [20], which is basically assigned the same thermal resistance for the same temperature differences at the upper and lower isotherm of the cycle. The results obtained for the Maximum Power Output are compared with those obtained by using the Maximum Efficient Power (MEP) and Maximum Power Density (MPD).

## 2. Modeling Methods and Results 

### 2.1. Agrawal’s Model

**Figure 1:**
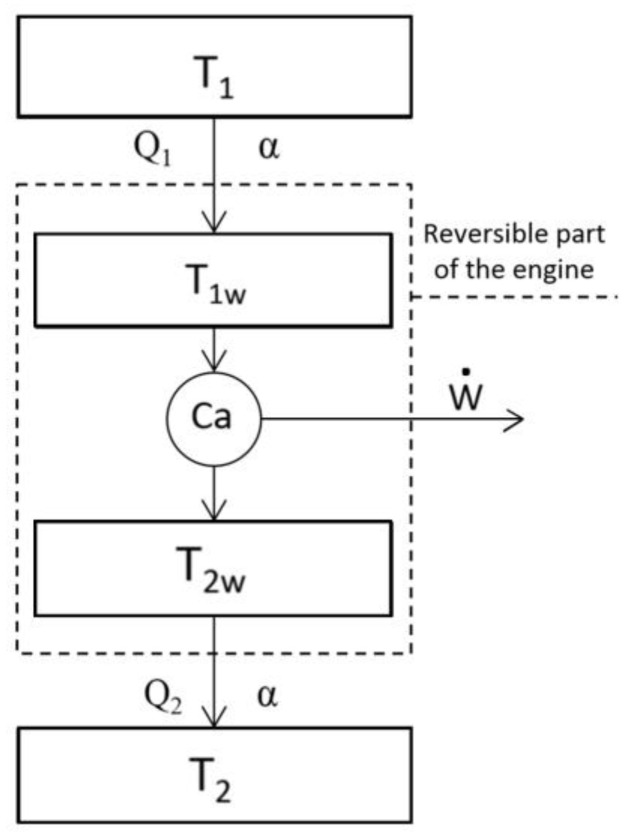
Simplified Agrawal’s model of a Curzon-Ahlborn engine.

The temperatures of hot and cold reservoirs and the temperatures of the working fluid substance for a Curzon-Ahlborn engine are related by:

(1) $$T_{1}\geq T_{1W}\geq T_{2W}\geq T_{2}$$

where $T_{1}$ is the temperature of the hot reservoir, $T_{2}$ is the temperature of the cold reservoir, and $T_{1W}$ and $T_{2W}$ are the working fluid temperatures of the heat engine at the hot and cold isotherms, respectively, as depicted in Figure 1. Also, in their famous paper, Curzon and Ahlborn [3] defined $x=T_{1}-T_{1W}$ and $y=T_{2W}-T_{2}$ as the temperature differences between thermal reservoirs and the isothermal branches of the internal cycle. Moreover, by using an algebraic method, Agrawal [20] proposed a simplified version of the Curzon-Ahlborn engine to help undergraduate students more easily understand the theory, in which, by assigning the same thermal resistance to the same temperature differences at the upper and the lower cycle isotherm, he obtained similar efficiency values to those obtained by Curzon-Ahlborn for real power plants. Furthermore, this model has remarkable similarities with other results obtained from finite time thermodynamics [21,22].

Now, by considering a Newton heat transfer law from the hot reservoir to the working fluid ($Q_{1}$) and from the working fluid to the cold reservoir ($Q_{2}$), we obtain:

$$\dot{Q_{i}}=\frac{dQ_{i}}{dt}=\alpha {\left(-1\right)}^{i-1}\left(T_{i}-T_{iW}\right),$$

where i=1 at the hot isotherm and i=2 at the cold isotherm of the cycle. For simplicity, an equal thermal conductance factor is considered in both heat transfer processes, α.

In accordance with the procedure followed by Agrawal [20] and repeated by Páez-Hernández et al. [21], the power output can be written as:

(2) $$\dot{W}=\;\alpha \frac{x\left(T_{1}-T_{2}-2x\right)}{T_{1}-T_{2}}.$$

### 2.2. Performance Using Different Criteria for the Newton Heat Transfer Law Case

#### 2.2.1. Maximum Power Output

In order to investigate the efficiency when a heat engine is working on a Maximum Power Output regime, let us consider that the temperatures of the engine working fluid, $T_{1W}$ and $T_{2W}$, work as a Carnot engine, so its efficiency is η=1−θ, where θ is the ratio of working temperatures, $\theta =T_{2W}/T_{1W}$. So, we obtain:

(3) $$\theta =\frac{T_{2}+y}{T_{1}-x}=\frac{T_{2}+x}{T_{1}-x},$$

where we are using the Agrawal assumption, in which the difference in temperatures is x=y.

Now, from the last equation, we can solve x obtaining:

(4) $$x=\frac{\theta T_{1}-T_{2}}{1+\theta },$$

which, substituted in Equation (2), leads to:

(5) $$\dot{W}=\alpha \frac{xT_{1}\left(\theta -\tau \right)\left(1-\theta \right)}{\left(1+\theta \right)}.$$

From the condition $d\dot{W}/d\theta =0$, the value of θ, where $\dot{W}$ has a maximum value, is:

(6) $$\theta_{MP}=\frac{1+3\tau }{3+\tau }.$$

Then, the efficiency at maximum power output regime yield is:

(7) $$\eta_{MP}=1-\frac{1+3\tau }{3+\tau },$$

and the maximum value of power output provides:

(8) $${\dot{W}}_{MP}=\alpha T_{1}\frac{{\left(1-\tau \right)}^{2}}{8\left(1+\tau \right)}.$$

#### 2.2.2. Maximum Power Density

Instead of using the Maximum Power Output and the efficiency in order to analyze the performance of thermodynamic cycles, recently Sahin et al. [13] introduced the Maximum Power Density criterion, which involves maximizing the ratio of the power to the maximum specific volume in the cycle. For the system that we are considering, the MPD can be defined as:

(9) $${\dot{W}}_{PD}=\frac{{\dot{W}}_{MPV}}{V},$$

where ${\dot{W}}_{PD}$ is the Power Density, ${\dot{W}}_{MPV}$ is the Maximum Power Output, and *V* is the Maximum Volume in the cycle.

Our next objective was to analyze in more detail the Power Density and establish a set of equations similar to Equations (7) and (8). Thus, following the process used in the previous section, we proceeded to maximize the Power Density.

Therefore, from Equation (9),we obtain:

(10) $${\dot{W}}_{PD}=\frac{\alpha T_{1}\left(\theta -\tau \right)\left(1-\theta \right)}{mR\theta \left(\theta +1\right)}$$

where we assume that the maximum volume in the cycle is an ideal gas, which can be written as:

(11) $$V=\frac{mRT_{2W}}{P_{min}},$$

where m is the mass of the working fluid, and R is the ideal gas constant. In this analysis, the minimum pressure $P_{min}$ in the cycle is taken to be constant. It is important to note that Equations (10) and (11) contain the *mR* constant parameter instead of *nR*, where *n* is the quantity of moles, as is common in classical equilibrium thermodynamics. This does not influence the analysis that is being performed because, during the processes of power derivation and normalization, this parameter disappears. In addition, as the mass and the number of molecules in a substance are proportional, this allows using these expressions without any conceptual problems.

Now, the condition $d{\dot{W}}_{MPD}/d\theta =0$ obtains the value of $\theta_{MPD}$, where ${\dot{W}}_{MPD}$ has a maximum value:

(12) $$\theta_{MPD}=\;\frac{\tau +\sqrt{2}\sqrt{\tau +\tau^{2}}}{2+\tau }.$$

Then, the efficiency of engine at maximum power regime yields:

(13) $$\eta_{MPD}=1-\frac{\tau +\sqrt{2}\sqrt{\tau +\tau^{2}}}{2+\tau }.$$

#### 2.2.3. Maximum Efficient Power

Let us analyze the Maximum Efficient Power regime. Yilmaz et al. [17] introduced the Maximum Efficient Power criterion as the multiplication of power output by cycle efficiency, obtaining:

(14) $${\dot{W}}_{EP}=\eta {\dot{W}}_{MP}.$$

As above, proceed to maximize the Efficient Power. Then, from Equation (13), we obtain:

(15) $${\dot{W}}_{EP}=\frac{\alpha T_{1}\left(\theta -\tau \right){\left(1-\theta \right)}^{2}}{{\left(\theta +1\right)}^{2}}.$$

The condition $d{\dot{W}}_{EP}/d\theta =0$ provides the value of $\theta_{MEP}$, where ${\dot{W}}_{MEP}$ has a maximum value, being:

(16) $$\theta_{MEP}=\;-2+\sqrt{5+4\tau }.$$

Then, the efficiency of the engine at MEP regime results in:

(17) $$\eta_{MPD}=3-\sqrt{5+4\tau }.$$

It is important to notice that in all the regimes considered above, we use a Newton heat transfer law for the interchange of energy between the heat reservoirs.

### 2.3. Performance Using Different Criteria for the Dulong-Petit Heat Transfer Law

Following the same procedure performed by Páez-Hernández et al. [21], we analyzed the engine shown in Figure 1, but considering a Dulong-Petit heat transfer law, whichis ${\dot{Q}}_{i}=dQ_{i}/dt=\alpha {\left(-1\right)}^{i-1}{\left(T_{i}-T_{iW}\right)}^{k}$, where α is the thermal conductance and k=5/4 is the exponent related to natural convection [23], with i=1 at the hot isotherm and i=2 at the cold isotherm of the cycle.

#### 2.3.1. Maximum Power Output

In order to investigate the efficiency when a heat engine is working at Maximum Power Output, let us consider that, for the Dulong-Petit heat transfer law, the MP, after some algebraic steps, is given by:

(18) $${\dot{W}}_{MP}^{DP}=\frac{\alpha T_{1}^{5/4}{\left(\theta -\tau \right)}^{5/4}\left(1-\theta \right)\left(1+\tau \right)}{{\left(\theta +1\right)}^{9/4}}.$$

Now, the condition ${\dot{W}}_{MP}^{DP}/d\theta =0$ produces the value $\theta_{MP}^{DP}$, where ${\dot{W}}_{MP}^{DP}$ has a maximum value being:

(19) $$\theta_{MP}^{DP}=\frac{5+13\tau }{13+5\tau },$$

where the superscript DP denotes that we are using the Dulong-Petit heat transfer law.

Then, the efficiency of the engine at Maximum Power Output with a Dulong-Petit heat yields:

(20) $$\eta_{MP}^{DP}=1-\frac{5+13\tau }{13+5\tau }.$$

#### 2.3.2. Maximum Power Density

Now, for the MPD, similar to the previous section, we proceed to maximize the power density for the cited heat transfer law. Therefore, it is easy to obtain:

(21) $${\dot{W}}_{PD}^{DP}=\frac{\alpha T_{1}^{5/4}{\left(\theta -\tau \right)}^{5/4}\left(1-\theta \right)\left(1+\tau \right)}{mR\left(1-\tau \right){\left(\theta +1\right)}^{5/4}}.$$

The condition $d\;{\dot{W}}_{PD}^{DP}/d\theta =0$ provides the value of $\theta_{MPD}^{DP}$, where ${\dot{W}}_{PD}^{DP}$ has a maximum value, being:

(22) $$\theta_{MPD}^{DP}=\;\frac{1}{8}\left(-9-\tau +\sqrt{161+162\tau +\tau^{2}}\right).$$

Then, the efficiency of engine at maximum power density regime yields:

(23) $$\eta_{MPD}^{DP}=\frac{1}{8}\left(17+\tau -\sqrt{\left(1+\tau \right)\left(161+\tau \right)}\right).$$

#### 2.3.3. Maximum Efficient Power

Let us analyze the Maximum Efficient Power regime. As before, we proceed to maximize the MEP. Then, using the definition given byYilmaz [17], we obtain:

(24) $${\dot{W}}_{EP}^{DP}=\frac{\alpha T_{1}^{5/4}{\left(\theta -\tau \right)}^{5/4}{\left(1-\theta \right)}^{2}\left(1+\tau \right)}{{\left(\theta +1\right)}^{\frac{9}{4}}}.$$

The condition $d\;{\dot{W}}_{EP}^{DP}/d\theta =0$ provides the value of $\theta_{MEP}^{DP}$, where ${\dot{W}}_{EP}^{DP}$ has a maximum value:

(25) $$\theta_{MEP}^{DP}=\;\frac{1}{8}\left(25+\tau -\sqrt{369+\tau \left(306+\tau \right)}\right).$$

Then, the efficiency of the engine at Maximum Efficient Power regimes is,

(26) $$\eta_{MEP}^{DP}=\frac{1}{8}\left(25+\tau -\sqrt{369+\tau \left(306+\tau \right)}\right).$$

## 3. Results

### 3.1. Newton Heat Transfer Law

**Figure 2:**
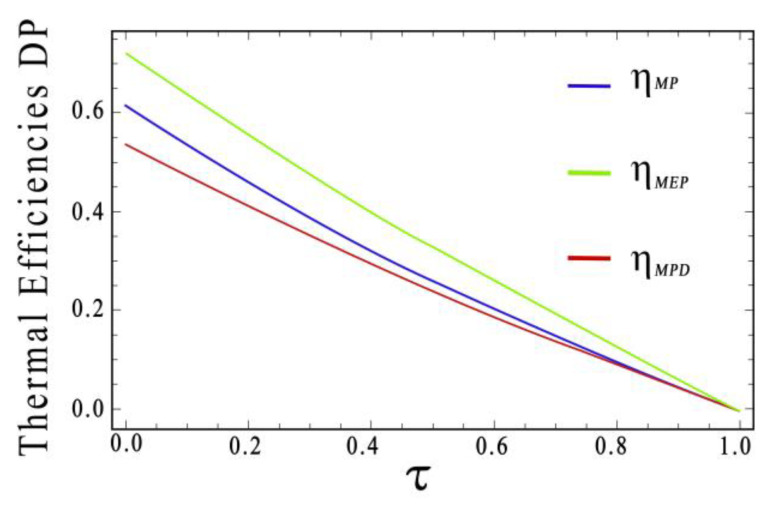
Comparison of thermal efficiencies at Maximum Power Output (MP), Maximum Efficient Power (MEP), and Maximum Power Density (MPD) regimes respect to efficiency (τ). In this case, we used a Newton heat transfer law.

A comparison of efficiencies $\eta_{MP}$, $\eta_{MPD}$, and $\eta_{MEP}$ for the Newton heat transfer law case is shown in Figure 2, in which, for certain values of τ(0<τ<1), we observe that $\eta_{MPD}>\eta_{MEP}>\eta_{MP}$ when τ≤0.19 and $\eta_{MEP}>\eta_{MPD}>\eta_{MP}$ when 0.19<τ<1. Additionally, the following statements are valid:

(27) $$\begin{array}{c} \eta_{MPD}>\eta_{MEP}>\eta_{MP},\;0<\tau <0.19\\ \eta_{MPD}=\eta_{MEP}>\eta_{MP},\;\tau \;=\;0.19\\ \begin{array}{c} \eta_{MEP}>\eta_{MPD}>\eta_{MP},\;0.19<\tau <1\\ \eta_{MEP}>\eta_{MPD}\approx \eta_{MP},\;0.68<\tau <1 \end{array} \end{array}$$

### 3.2. Dulong-Petit Heat Transfer Law Case

**Figure 3:**
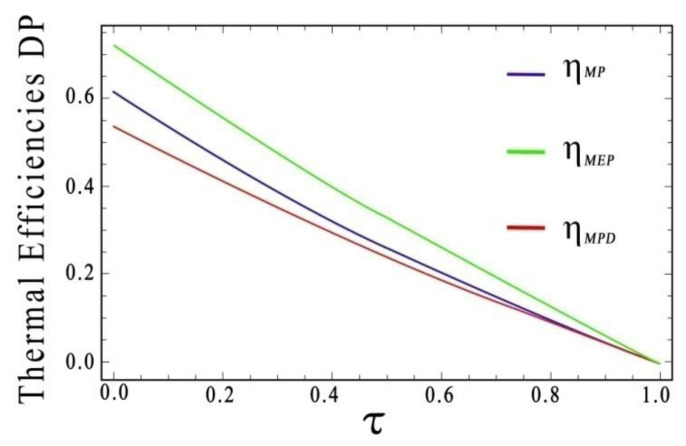
Comparison of thermal efficiencies at MP, MPD, and MEP regimes respect to τ. In this case, we used a Dulong-Petit heat transfer law.

Now, using the same procedure as in the previous section, a comparison of efficiencies at MP, MPD, and MEP is shown in Figure 3, in which a similar relation of the efficiencies, as shown in Equation (27), is fulfilled:

(28) $$\begin{array}{c} \eta_{MEP}>\eta_{MP}\geq \eta_{MPD},\;0<\tau <1\\ \;\eta_{MEP}>\left(\eta_{MPD}=\eta_{MP}\right)\;0.68<\tau <1 \end{array}$$

**Figure 4:**
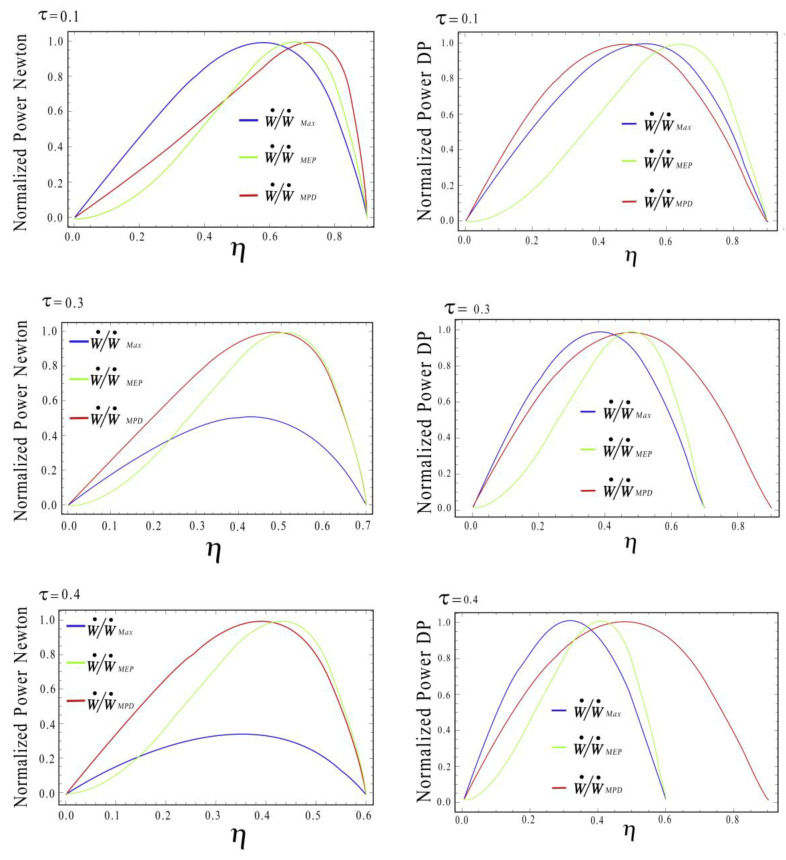
Comparison of thermal efficiencies at MP, MPD and MEP regimes respect to thermal efficiency, (**left**) Newton heat transfer law and (**right**) Dulong-Petit heat transfer law; for different values of τ.

Now, in order to compare the different operation regimes depicted in Figure 4, we calculated the normalized dimensionless power $\left(\dot{W}/{\dot{W}}_{Max}\right)$, considering ${\dot{W}}_{Max}$ for each case, i.e., the MP, MPD, and MEP versus the efficiency. Notice that in Figure 4, we use some arbitrary values for the efficiency (τ=0.1, 0.3, 0.4), and on the left side of Figure 4, we depict the cases for Newton heat transfer law and right side the Dulong-Petit heat transfer law.

## 4. Conclusions 

This paper presents a finite-time thermodynamic optimization based on three different optimization criteria—Maximum Power Output (MP), Maximum Efficient Power (MEP) and Maximum Power Density (MPD)—for a simplified Curzon-Alhborn engine proposed by Agrawal [20]. Despite the model being very simple, it captures the behavior determined by Yilmaz et al. [24], in the sense that, for a Newton heat transfer law, we observed that $\eta_{MPD}>\eta_{MEP}>\eta_{MP}$ when τ≤0.19, and $\eta_{MEP}>\eta_{MPD}>\eta_{MP}$ when 0.19<τ<1. Moreover, when τ=0.19, $\eta_{MPD}=\eta_{MEP}$. This is interesting because the same result was obtained by Yilmaz et al. [24] but using a more robust model that even included irreversibilities. The latter shows that the oversimplified model proposed by Agrawal could be used to model some real heat engines. When τ=0.68, $\eta_{MPD}=\eta_{MP}$ but is less than $\eta_{MEP}$. This result is not the same as that obtained by Yilmaz et al. [24] but is close to the one reported. In our case, additionally, we changed the heat transfer law, so instead of using a Newton law, we proposed a Dulong-Petit heat transfer law. For this case, we observed that $\eta_{MEP}>\eta_{MPD}>\eta_{MP}$ when 0<τ≤0.68 and $\eta_{MEP}>\eta_{MPD}\geq \eta_{MP}$ when 0.68<τ<1.

The behavior of the Maximum Power Density can be explained by involving different operation parameters of a thermal engine related to its design restrictions. This depends on the high power or high efficiency of the heat engine. Notably, when the Dulong-Petit heat transfer law was used, the interval $\eta_{MEP}>\eta_{MPD}>\eta_{MP}$ was greater than in the Newton heat transfer law case.

The above evaluation can be seen more clearly in Figure 4, where, for a specific τ value, the thermal efficiency at the MEP ($\eta_{MEP}$) condition was greater than the other conditions, MPD and MP. However, if the parameter τ changed, the behavior of the efficiency also changed. Moreover, some authors [25,26] showed that a good trade-off between the engine performance and its dynamic behavior occurs when 0.32≤τ≤0.64, providing important guidance when real power plants are designed. This indicates that τ plays an important role in engine performance as other authors have stated [27,28].
